# Ecological memory and relocation decisions in fungal mycelial networks: responses to quantity and location of new resources

**DOI:** 10.1038/s41396-019-0536-3

**Published:** 2019-10-18

**Authors:** Yu Fukasawa, Melanie Savoury, Lynne Boddy

**Affiliations:** 1 School of Biosciences, Cardiff University, Museum Avenue, Cardiff, CF10 3AX UK; 2 Graduate School of Agricultural Science, Tohoku University, 232-3 Yomogida, Naruko, Osaki, Miyagi, 989-6711 Japan

**Keywords:** Fungal ecology, Microbial ecology

## Abstract

Saprotrophic cord-forming basidiomycetes, with their mycelial networks at the soil/litter interface on the forest floor, play a major role in wood decomposition and nutrient cycling/relocation. Many studies have investigated foraging behaviour of their mycelium, but there is little information on their intelligence. Here, we investigate the effects of relative size of inoculum wood and new wood resource (bait) on the decision of a mycelium to remain in, or migrate from, inoculum to bait using *Phanerochaete velutina* as a model. Experiments allowed mycelium to grow from an inoculum across the surface of a soil microcosm where it encountered a new wood bait. After colonisation of the bait, the original inoculum was moved to a tray of fresh soil to determine whether the fungus was still able to grow out. This also allowed us to test the mycelium’s memory of growth direction. When inocula were transferred to new soil, there was regrowth from 67% of the inocula, and a threshold bait size acted as a cue for the mycelium’s decision to migrate for a final time, rather than a threshold of relative size of inoculum: bait. There was greater regrowth from the side that originally faced the new bait, implying memory of growth direction.

## Introduction

Fungi are vital agents for organic matter decomposition, and carbon and nutrient cycling in forest ecosystems by virtue of their huge biomass, enzymatic ability, and efficient translocation of carbon and nutrients by mycelial networks [1]. Cord-forming basidiomycetes are particularly important due to the persistent linear organs that they produce—cords. Cords are aggregations of many parallel-aligned hyphae, which are often differentiated internally, forming large networks at the interface of the litter layer and soil horizon in the forest floor, translocating carbon and nutrients efficiently [2–4]. Cord-forming basidiomycetes colonise, and link together, many different plant litter components within its cord network, from leaf litter and small twigs to large, fallen tree trunks [1]. They are abundant on the forest floor [1], often occupying large areas and being long-lived [5–8]. A better understanding of developmental cues, nutrient translocation and the mechanisms of network sustainability are essential for understanding cycling and redistribution of carbon and nutrients on the forest floor.

The behavior of cord-forming mycelium has been well-studied using soil tray microcosm experiments [9, 10]. When a wood block colonised by a cord-forming basidiomycete is placed as an inoculum on the surface of compressed soil, mycelium grows out from the inoculum onto the soil, colonising any new resources that it encounters. If a newly encountered resource (bait) is sufficiently large compared with the inoculum, connecting cords thicken and non-connected mycelium regresses, resulting in a strong connection between inoculum and bait. Nutrient translocation between resources, via cords, can occur in both directions but extent and timing depends on relative size and decay stage of them, probably reflecting a ‘source and sink’ relationship [2, 11]. Similar patterns are seen on the forest floor [10], and there is evidence that mycelium sometimes completely abandons small resources [12]. The latter phenomenon has not been investigated experimentally.

Foraging and migration behaviour has also been studied using myxomycete plasmodia which, though unicellular, have a superficially similar body design to fungal mycelia, are both heterotrophs, feeding by extracellular digestion, although plasmodia also use phagocytosis [13]. From numerous studies on the model species *Physarum polycephalum*, it is known that myxomycete plasmodia can optimise their network structure to connect separately located multiple resources, avoid unfavorable areas [14, 15] and solve mazes [16]. They can remember their past activities to avoid previously explored areas, but can decide to enter unfavorable areas if there is no other choice [17], and the time to leave old food sources is determined heuristically [18]. Therefore, plasmodia of myxomycetes are now considered to have intelligence and cognitive abilities even though they have no brain, central nervous system, nor neural networks [18]. If fungal mycelial cord networks have similar intelligence, it will completely change our understanding of carbon and nutrient cycling on the forest floor.

The aims of this study were to (1) determine what conditions make a fungal mycelium decide to make its final move from an old inoculum to a new wood resource (bait), and (2) test whether a fungal mycelium within an inoculum remembers the direction of a new resource bait to which it had been connected, if the cord connection between the inoculum and bait was completely destroyed. We hypothesised that relative size of inoculum and bait wood blocks would affect their decision to move and memory of direction of the bait. We used a soil tray microcosm and a saprotrophic cord-forming basidiomycete *Phanerochaete velutina* (DC.) P. Karst. as a model system. This fungus is common in UK forests [6] and is one of the most well-studied species in the research field of mycelial network behaviour [1, 9, 10].

## Materials and methods

### Fungal culturing and inoculum preparation

Kiln dried beech (*Fagus sylvatica*) wood was cut into blocks of three sizes: 0.5 × 1 × 1 cm (0.5 cm^3^), 2 × 1 × 1 cm (2 cm^3^), 2 × 2 × 1 cm (4 cm^3^). Blocks were soaked overnight in DH_2_O prior to use and then autoclaved at 121 °C for 20 min in double, sealed autoclave bags. The process was repeated three times with 1 day intervals. Sterilised wood blocks were placed onto cultures of *P. velutina* (Cardiff University Collection) which was grown on 0.5% malt extract agar (MEA; 5 g Lab M malt extract, 15 g Lab M agar no. 2) in non-vented 14 cm-diameter Petri dishes (2-cm thick). Plates were sealed with Parafilm®, (Bemis Company Inc., Oshkosh, USA) and incubated in the dark at 20 °C for 3 months before use.

### Microcosm preparation and inoculation

Soil was collected from the top 10 cm in a deciduous forest in Tintern Monmouthshire (51.6980 N, 2.6814 W). After sieving on site (10 mm mesh), the soil was air-dried, sieved through a 2-mm mesh and frozen at −18 °C for 48 h. Soil (200 g) was rehydrated with 300 ml DH_2_O (to give −0.012 MPa) and transferred to 24 × 24 cm bioassay dishes, smoothed and compacted to about 5 mm depth. A wood block, from which surface mycelia and excess agar had been removed using a razor blade, was placed centrally, 5 cm from one side of each soil tray. When mycelia had extended 6 cm from the inoculum in 50% of the trays, a new beech wood block (bait) prepared and sterilized as described above, but not inoculated with fungi, was placed at the margin of the mycelium towards the middle of the tray. Six sizes of bait wood blocks [0.5 × 1 × 1 cm (0.5 cm^3^), 1 × 1 × 1 cm (1 cm^3^), 2 × 1 × 1 cm (2 cm^3^), 2 × 2 × 1 cm (4 cm^3^), 4 × 4 × 1 cm (16 cm^3^), and 6 × 6 × 1 cm (36 cm^3^)] were tested in all combinations with the three inoculum sizes (i.e. 18 combinations in total). Ten replicates were made for each combination (i.e. a total of 180 tray microcosms) (Fig. S1).

### Microcosm incubation

After set-up, each tray was weighed, and lost water was replaced every week by spraying DH_2_O evenly across the soil until each tray reached its original mass. Trays were stacked and sealed in polythene bags to reduce water loss, and incubated at 20 °C and 70% humidity in the dark for 48 days [period I].

After period I, inoculum wood blocks were retrieved, cleaned of surface mycelia, and placed centrally onto new soil trays freshly prepared as described above. The trays were further incubated at 20 °C and 70% humidity for 8 days [period II], and the presence and location of outgrowing myclium was recorded. The systems were incubated for a further 8 days and then rerecord, but there was little change in results, so we only analysed the 8th day data. Previous studies on *P. velutina*, and our personal observation in period I in the present study, showed that there is substantial hyphal growth from inoculum at 2–5 days after placing onto the soil [19, 20], suggesting that 8 days are sufficient to check for outgrowth from the inoculum.

Trays were randomly repositioned every 3 days during incubation (in both periods I and II) to avoid possible effects of orientation and location within the CT room on the direction of hyphal growth. Trays were photographed when the baits were added to the tray, and at the end of incubation period I and II, using a Nikon Coolpix P80 camera, mounted on a stand at a height of 46 cm, and in the same light conditions to ensure consistency.

### Image analysis

**Fig. 1:**
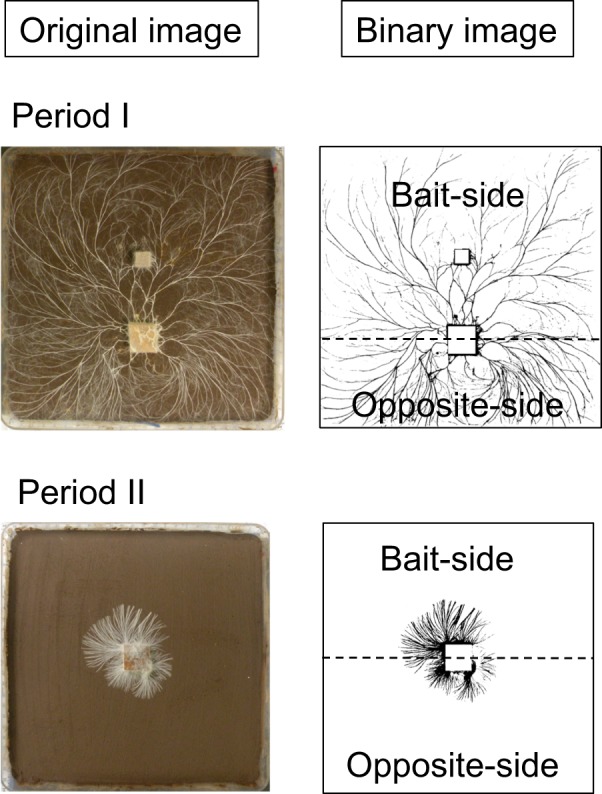
Measurement of hyphal coverage on soil. Original images were converted into binary images by ImageJ (National Institute of Health, colour threshold = 160). To evaluate hyphal growth on the bait-side and opposite-side of the inoculum wood block, each image was split into two parts at the centre line of the inoculum wood block (dashed lines)

Images were analysed using ImageJ (National Institute of Health, USA). A 2-cm calibration line was drawn electronically using a ruler next to each tray. The edge of the soil tray and wood block were removed by windowing, and the resulting image converted to black and white binary with a manually set threshold. The mycelia and soil were indicated by black and white pixels, respectively, allowing hyphal coverage (cm^2^) to be determined. Given the difference in bait size, we calculated hyphal density on soil plates by dividing hyphal coverage (i.e. pixel count) by soil area to be compared between different bait size experiments. To compare mycelial growth towards and away from the bait, each image was split into two at the center line of the inoculum wood block (Fig. 1). Hyphal coverage ratio of bait-side and opposite-side were calculated by dividing hyphal coverage of the side closest to the new resource (termed bait-side) and the opposite-side by the hyphal coverage of the whole mycelium.

### Statistical analysis

Hyphal density (pixel count per unit soil area) in period I and hyphal coverage in period II were compared across experiments within treatments with the same inoculum size by Tukey’s pairwise comparison (*P* < 0.05). Hyphal coverage ratio between bait-side and opposite-side were compared by Wilcoxon rank-sum test.

Effects of inoculum size (*Inoc*), bait size (*Bait*), distance between inoculum and bait (*D*), and interaction between inoculum and bait sizes (*Inoc* × *Bait*) on hyphal regrowth in period II were evaluated using generalised linear models (GLMs). The first model (GLM_1) was applied to explain the presence/absence of regrowth in period II (*GIIcount*). The second model (GLM_2) evaluated the effects of four predictor variables in GLM_1 plus bait-side growth ratio in period I (*GIbait*) on bait-side growth ratio from inoculum in period II (*GIIbait*). A binomial distribution error was assumed and a logit link function was used in GLM_1, whereas Gaussian distribution was assumed and an identity link function was used in GLM_2. The model descriptions are as follows:

$$GIIcount_{i} \sim 	 {\mathrm{Binomial}}\left( \mu_{\mathrm{i}} \right),\\ logit\left(\mu_i \right) = 	 \beta _0 + Inoc_i + Bait_i + D_i + Inoc_i \\ 	 \times Bait_i\,\hbox{---}\,{\mathrm{GLM}}\_{\mathrm{1}},\\ GIIbait_i \sim 	{\mathrm{Gaussian}}\left( \mu_{i} \right), \\ identity\left(\mu _i \right) = 	 \beta _0 + Inoc_i + Bait_i + D_i + Inoc_i \times Bait_i \\ 	 + GIbait_i\hbox{---}{\mathrm{GLM}}\_{2},$$

where *β*_*0*_ is the intercept and *i* stands for individual soil microcosm. In the present study, it inevitably happens that the distance between inoculum block and bait block differs slightly among the treatments (Fig. S2). We were not interested in distance effects, but included it in the model to check that it had no effect on the results. We included an interaction term between inoculum size and bait size in the models because we hypothesized that relative size of inoculum and bait determines the growth response of fungi in the microcosm. For model simplicity to keep statistical power, we did not include interaction terms between distance and wood sizes.

For both GLM_1 and GLM_2, the best models were selected based on the Akaike information criterion by backward stepwise selection. The coefficients of the best models were exponentiated to obtain odds ratios (for GLM_1) or risk ratios (for GLM_2). Ratios >1 indicated that the explanatory variable had a positive effect on the presence/absence of regrowth in period II (GLM_1) or bait-side hyphal coverage in period II (GLM_2), while ratios <1 indicated negative effects; the difference from one indicated the magnitude of the effect. The level of collinearity between predictor variables was checked by calculating the variance inflation factor (VIF): all VIF values were <3, indicating low levels of multicollinearity in the models.

All statistical analyses were conducted in R 3.5.0 (R core team, 2018) using the DAAG [21] and MASS [22] packages. A power analysis was performed using G*Power software [23], which confirmed that the sample size (*n* = 180) was sufficient to test the effects of the five variables (including interaction terms) on hyphal regrowth in period II.

## Results

### Growth characteristics in period I

There was no significant difference (*P* > 0.05) in hyphal area ratio between mycelium growing out from the inoculum on the bait-side and the opposite-side at the time when the baits were added (Fig. S3), suggesting that there was no preference in hyphal growth direction before baiting. Colonisation of baits by *P. velutina* hyphae occurred in all soil trays (Fig. S4).

**Fig. 2:**
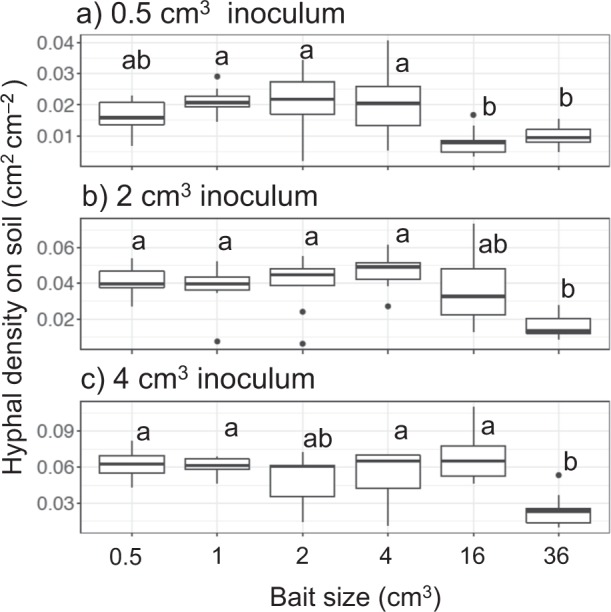
Hyphal density (cm^2^ cm^−2^) on soil in laboratory microcosms growing from **a** 0.5 cm^3^, **b** 2 cm^3^, and **c** 4 cm^3^ inocula 48 days after a new bait resource was added. Different letters on each box show significant (*P* < 0.05) difference across the six bait sizes (Nemenyi-tests, Tukey: *N* = 10). Note that the *y* axes have different scales

**Fig. 3:**
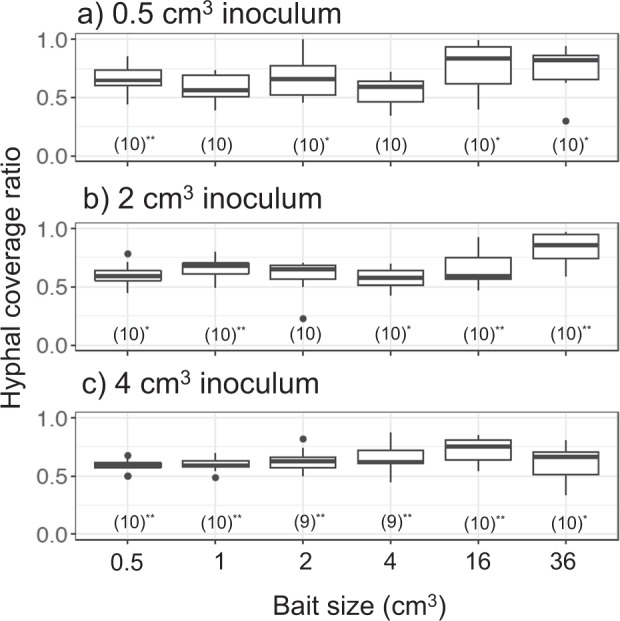
Bait-side hyphal coverage ratio against whole mycelium coverage (bait-side + opposite-side) of **a** 0.5 cm^3^, **b** 2 cm^3^, and **c** 4 cm^3^ inocula 48 days after baiting in period I. Values in parenthesis are the number of replicates for each experiment, with asterisks indicating a significant difference from 0.5 (Wilcoxon rank sum test: *P < 0.05; **P < 0.001)

As the soil area available for mycelial colonization is inherently different in soil trays with different-sized baits, we compared hyphal density on soil in period I across experiments within the same inoculum size. Forty eight days after baiting, hyphal coverage was usually significantly less in mycelial systems with the largest (36 cm^3^ and sometimes 16 cm^3^) baits than that coupled with the smaller (1, 2, and 4 cm^3^) baits (Figs. 2; S4). Mycelium on the soil in the area of the inoculum wood block, but not connected to the bait, often died back in systems with largest baits (Fig. S4c), but not in systems with smaller baits (Fig. S4). In most of the combinations, except for 0.5 cm^3^ inoculum coupled with 4 cm^3^ bait, hyphal area ratio on the bait-side was significantly larger than that of the opposite-side (Fig. 3).

### Regrowth in period II

**Fig. 4:**
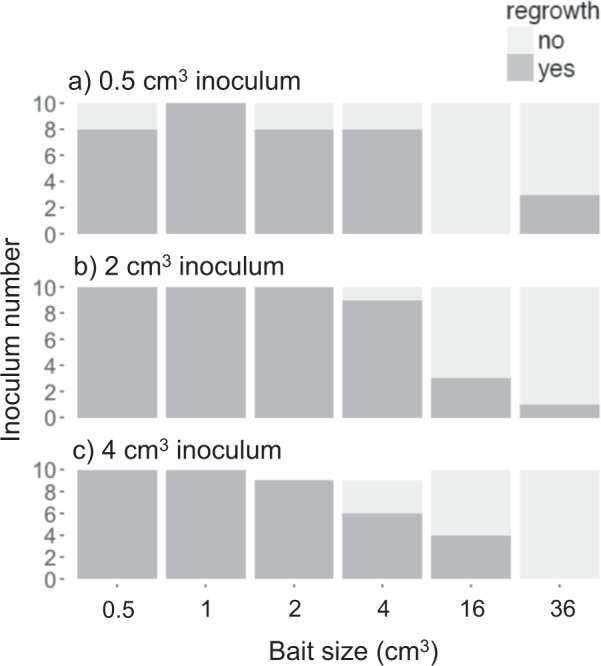
Frequency of **a** 0.5 cm^3^, **b** 2 cm^3^, and **c** 4 cm^3^ inocula with/without hyphal regrowth 8 days after inocula had been moved to new soil trays, depending on the size of bait encountered in period I

Inocula coupled with 16 and 36 cm^3^ baits seldom showed regrowth in period II regardless of the inoculum size (Fig. 4). All of the 2 and 4 cm^3^ inocula whose mycelium joined to small baits (0.5, 1, and 2 cm^3^) showed regrowth, although some of the 0.5 cm^3^ inocula linked to 0.5 and 2 cm^3^ baits did not show regrowth. Bait volume had a less predictable effect on regrowth from small inocula than from larger inocula. Among the four variables tested in GLM_1, volumes of inoculum and bait, and their interaction term, were significantly related to occurrence of regrowth, and were selected as factors in the best model (Table 1). The inoculum volume had a strong positive association, and the bait volume had a negative association with occurrence of regrowth from the inoculum.

**Table 1:** GLM_1 results explaining presence/absence of regrowth from the inoculum 8 days after being moved to a new soil tray

Variable	Estimate	Odds ratio
Inoculum volume	0.56*	1.75
Bait volume	−0.08*	0.92
Inoculum:Bait volume	−0.04*	0.96
Distance	–	–

Distance between inoculum and bait was not selected in the best model according to lowest AIC

*P < 0.05

**Fig. 5:**
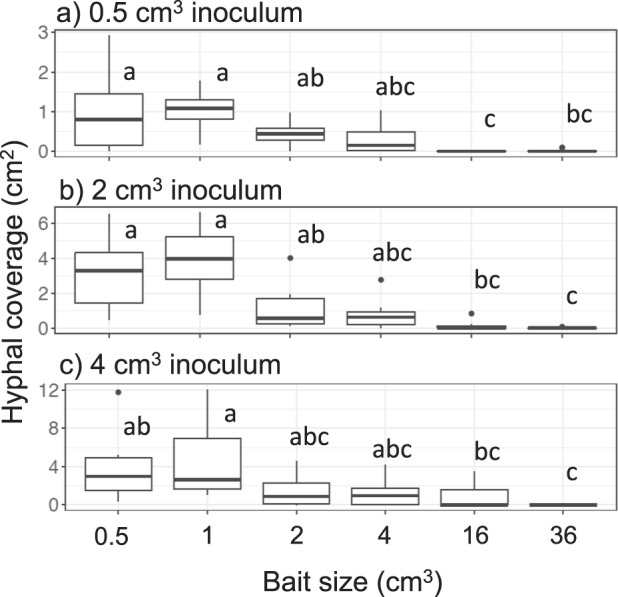
Hyphal coverage (cm^2^) of mycelia extending from **a** 0.5 cm^3^, **b** 2 cm^3^, and **c** 4 cm^3^ inocula 8 days after they had been moved to new soil trays. Different letters on each box indicate significant (*P* < 0.05) difference across the six bait sizes (Nemenyi-tests, Tukey: *N* = 10). Note that the *y* axes have different scales

**Fig. 6:**
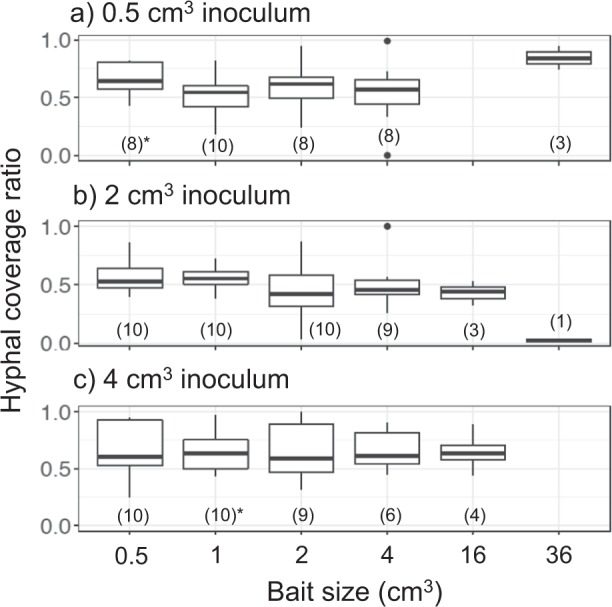
Bait-side hyphal coverage ratio against whole mycelium coverage (bait-side + opposite side) in period II of **a** 0.5 cm^3^, **b** 2 cm^3^, and **c** 4 cm^3^ inocula 8 days after they had been moved to new soil trays. Values in parenthesis are the number of replicates for each experiment, with asterisks indicating significant difference from 0.5 (Wilcoxon rank sum test: *P < 0.05)

Similar to period I, hyphal coverage in period II was usually significantly less in mycelial systems linked to larger baits than in those linked to smaller baits (Fig. 5). Hyphal area ratio of the bait-side of the inoculum was significantly larger than that of the opposite-side growing from 0.5 cm^3^ inocula previously linked to 0.5 cm^3^ bait, and from 4 cm^3^ inocula previously linked to 1 cm^3^ bait (Fig. 6). Among the five variables tested in GLM_2, inoculum volume and bait-side hyphal growth ratio in period I had significant positive associations with bait-side hyphal growth ratio in period II (Table 2). Among them, bait-side hyphal growth ratio in period I had a particularly large risk ratio, indicating a strong effect. Bait volume, interaction between inoculum and bait volume, and distance between inoculum and bait were also selected in the best model, but their associations with bait-side growth in period II were not significant.

**Table 2:** GLM_2 results explaining hyphal area ratio in bait-side of the inoculum 8 days after being moved to a new soil tray

Variable	Estimate	Risk ratio
Inoculum volume	0.03*	1.03
Bait-side ratio in period I	0.50**	1.64
Bait volume	0.003	1.00
Inoculum:Bait volume	−0.003	1.00
Distance	−0.02	0.98

All five variables were selected in the best model according to lowest AIC

*P < 0.05; **P < 0.01

## Discussion

### Mycelial decision to migrate

We have shown that when mycelia of *P. velutina* grew from inoculum wood blocks and colonised new larger bait wood resources, if the interconnection was subsequently severed, mycelium was often no longer able to grow out of the original inoculum. We did not attempt to reisolate the fungus from the original inoculum, so we cannot be certain whether the fungus had completely lost its viability within the original inoculum. However, the observations certainly suggest more or less complete migration of active mycelial resources from the inoculum to the bait.

As predicted, larger baits induced complete migration more frequently than small baits. Interestingly, the threshold volume of the bait that induced dramatic change in frequency of regrowth from original inoculum was somewhere between 4 and 16 cm^3^ regardless of the inoculum volume (which ranged from 0.5 to 4 cm^3^). This suggests that the primary factor affecting a mycelial decision to migrate completely to a new resource is actual volume of the new resource rather than the relative size of new resource to original inoculum, at least within the range of wood volume tested in the present study. This may seem counter-intuitive, as the mycelial outgrowth pattern was determined only by the nutritional status of the wood resources, because a larger inoculum contains a larger amount of carbon available to mycelium compared with a smaller inoculum [19, 24]. However, since a mycelium is an integrated system, coordinated resource allocation within a mycelium may explain this behavior. *P. velutina* mycelium tends to allocate more phosphorus to large wood resources than to smaller ones [19, 25], suggesting that there is a relatively larger nutritional cost for early colonisation of a larger resource than of a smaller one. This may also explain why mycelial migration from large inocula to the baits is determined by a relatively strict bait volume, whereas this was not the case with migration from small inocula. Given a larger cost to maintain a mycelial presence in large inocula than in smaller inocula, the decision to keep or discard a large inoculum after finding new large resources may be strongly determined by nutritional economy of the mycelium, whereas with small inocula the decision to keep or discard the original inoculum may be more stochastic.

Although decay rates of wood blocks were not measured in the present study, size-dependent wood decay rate may also affect the fungal decision of whether or not to migrate. Since decay rate of smaller wood particles is generally faster than larger ones [26], the more rapidly decreasing energy content of smaller wood blocks may cause the mycelium to completely migrate to new resources sooner than from larger ones. Thus, incubation periods longer than 48 days may alter the relationships between migration and wood size.

Microbial competitors in soil may also affect the decision of whether or not to migrate. Since the soil used in the microcosm was unsterilised, the focal fungi have to defend their wood blocks from a variety of microbial competitors in soil, which has an energetic cost. Smaller wood territory is more difficult to defend against mycelia occupying larger territory [27, 28], supporting our results showing that *P. velutina* mycelium left the smallest inoculum more often than large baits.

It is not clear why the mycelial decision to migrate depended on a certain range of bait size, but not on relative size of bait to inoculum. A possible reason is the limitation in maximum possible size of mycelium in the microcosms regardless of the volume of wood resources within [19, 29]. Since wood is relatively poor in mineral nutrients, e.g. nitrogen and phosphorus [30], most of the nutrients necessary for initial mycelial establishment within new woody resources will be translocated in the foraging mycelium, originating from soil, stored or recycled within resources [4, 19, 20, 25]. To maintain the carbon to nutrient ratio of mycelium, the amount of carbon source (wood block) available for a mycelium is determined by nutrient acquisition, which largely depends on the size of mycelium [4]. In this context, the threshold volume of a bait that would make a mycelium decide to migrate completely would change according to the size of microcosm, and must be larger in the field where *P. velutina* mycelium colonises larger coarse woody debris [6]. The small size of microcosms may also be the reason why the distance between inoculum and bait wood blocks did not affect the results in the present study. *P. velutina* is known as a ‘long-range forager’ [10], often forming cord networks extending over many meters [6, 12]. Cords of *P. velutina* can translocate phosphorus at least 75 cm within 5 days in the field [31] and probably very much further and faster, given carbon transfer to 18 cm distance from inoculum within 20 min in laboratory microcosms [29]. These effects of microcosm size and incubation time on fungal decisions should be tested in the future. Furthermore, relationships between fungal decision and sizes of inoculum and bait wood blocks should also be tested in more detail using wood blocks with a wider size range and narrower size intervals, because the size range of wood blocks were not evenly distributed in this study. Although the use of unsterilised soil provided a realistic scenario, the systems were much simplified with various stresses (such as fluctuating temperature and moisture) and disturbance agents (such as soil arthropods) prevented. These may also affect the nutritional economy of the mycelium and thus alter the migration threshold in natural ecosystems.

### Mycelial memory of direction of growth

Reallocation of mycelial biomass and mycelial growth in the direction of the bait, as seen in period I, is in line with previous findings (reviewed in [10]). The completely novel finding is the dominant regrowth, in period II, from the inoculum side that had originally been joined to the newly colonised resource in period I. This is a kind of memory of mycelial systems for spatial navigation and is likely to be advantageous for quickly repairing damaged network connections, by regrowing towards self and growing in a direction where resources have been found to be plentiful. In other circumstances, exploring a new area and avoiding effort in a previously recently explored region, might increase the chance of finding new resources. With regard to mechanisms, larger and newer wood baits have a greater flux of nutrients towards them [25, 32, 33], probably attributable to the large metabolic demand of an invasive mycelium in newly-colonized wood [24]. Previous studies found that destructive disturbance of cord networks of *P. velutina*, removing several baits [34] or severing cords [35], caused polarised growth in the undisturbed direction. Such polarisation may be attributable to undisturbed hyphae forming a ‘dominant-sink’ for translocation within the mycelial system.

In the present study, it is not appropriate to say that the mycelium remembered the direction of the bait since the effects of bait itself and difference in soil area between bait-side and opposite-side could not be evaluated separately. Further, absence of a second control comprising systems without an added bait wood block did not allow us to completely evaluate the effects of bait wood blocks on the hyphal growth in period II. However, the design allowed us to confirm that mycelia had no preference in growth direction before addition of bait, and thus we can say that there was memory of the predominant direction in which the mycelium developed. Previous studies have categorised the biotic mechanisms of memory in organisms (or swarms) without (central) nervous systems into two types [36]: (1) external memory, which detects signals deposited in the environment; (2) somatic memory, achieved by storage of both epigenetic and/or non-genetic changes of cell physiology. An example of (1) is foraging plasmodia of slime moulds which avoid areas that have previously been explored by detecting deposited extracellular slime [17]. Foraging ants, on the other hand, use trace pheromones to attract (rather than repel) conspecific individuals to trails which allows sharing information about food, nest or mate location [37]. However, such external memory is not likely to be the case in the present study because the inocula were moved to completely fresh soil trays without any deposits from previous activity. Furthermore, previous disturbance studies without changing the soil tray showed no evidence of positive or negative effects of the area previously covered with mycelium [35].

Evidence for the possibility of (2), somatic memory, in fungi was provided in a recent study on *Saccharomyces cerevisiae*, which showed that epigenetic transcriptomic change in mother cells that had experienced environmental change could be transferred to daughter cells, which had not experienced the environmental change [38]. Further, non-genetic changes induced by the environment, such as chemical concentrations and bioelectricity within a single cell [39–42], or in networks across multiple cells in multicellular organisms [43], can also act to maintain memories of polarised growth or habituation if cells were stored after disturbance, dormancy, or regenerations. The third possibility for explaining preferential bait-side regrowth in the present study is a carry-over effect of differential distribution of mycelium within the inoculum wood block, without any physiological change in the mycelium with more mycelium in the inoculum on the side nearest the bait. It is also valid to consider this to be a part of a memory mechanism because the mechanisms of memory in the human brain includes this kind of non-physiological, non-epigenetic phenotypic level change in neuronal network structure [44]. However, although we appreciate that there may be semantic conflicts in the concept of non-neuronal memory among scientists as it is a novel and developing study field [36], we believe that recognizing such a carry-over effect as a kind of memory is a first step in the study of non-neuronal intelligence (in the words of Solé et al. [36] ‘liquid brain’) in a broader sense. Determining which of the above-mentioned mechanisms are involved in a mycelial memory in our system is the next experimental challenge.

It is interesting that larger inocula tended to remember their direction of growth better than smaller inocula in the present study. Previous studies on *P. velutina* also reported that mycelium growing from large (8 cm^3^) wood blocks showed stronger polarity in nutrient transfer [24] and growth [19] compared with mycelium growing from smaller wood blocks. However, the mechanisms of polarity and memory in fungal mycelium have been poorly explored and are a challenging topic for the future. Whatever the mechanisms involved in the memory of mycelium, the results presented here show that mycelium of *P. velutina* remembered its growth direction after the complete removal of outgrowing hyphae from wood inocula. Recognizing that fungal mycelium has a primitive intelligence with decision-making ability and memory is an important step towards understanding mycelial foraging behaviour, with consequences for carbon and nutrient dynamics on the forest floor.

## Supplementary information

Figure S1

Figure S2

Figure S3

Figure S4

Dataset 1
