# Supplementary material for: Ecological memory and relocation decisions in fungal mycelial networks: responses to quantity and location of new resources
Source: ISME J. 2019 Oct 18;14(2):380–8. doi: 10.1038/s41396-019-0536-3 (PMC6976561; doi:10.1038/s41396-019-0536-3)
Supplement: Supplementary file 1 — Figure S1 [file 41396_2019_536_MOESM1_ESM.pdf]

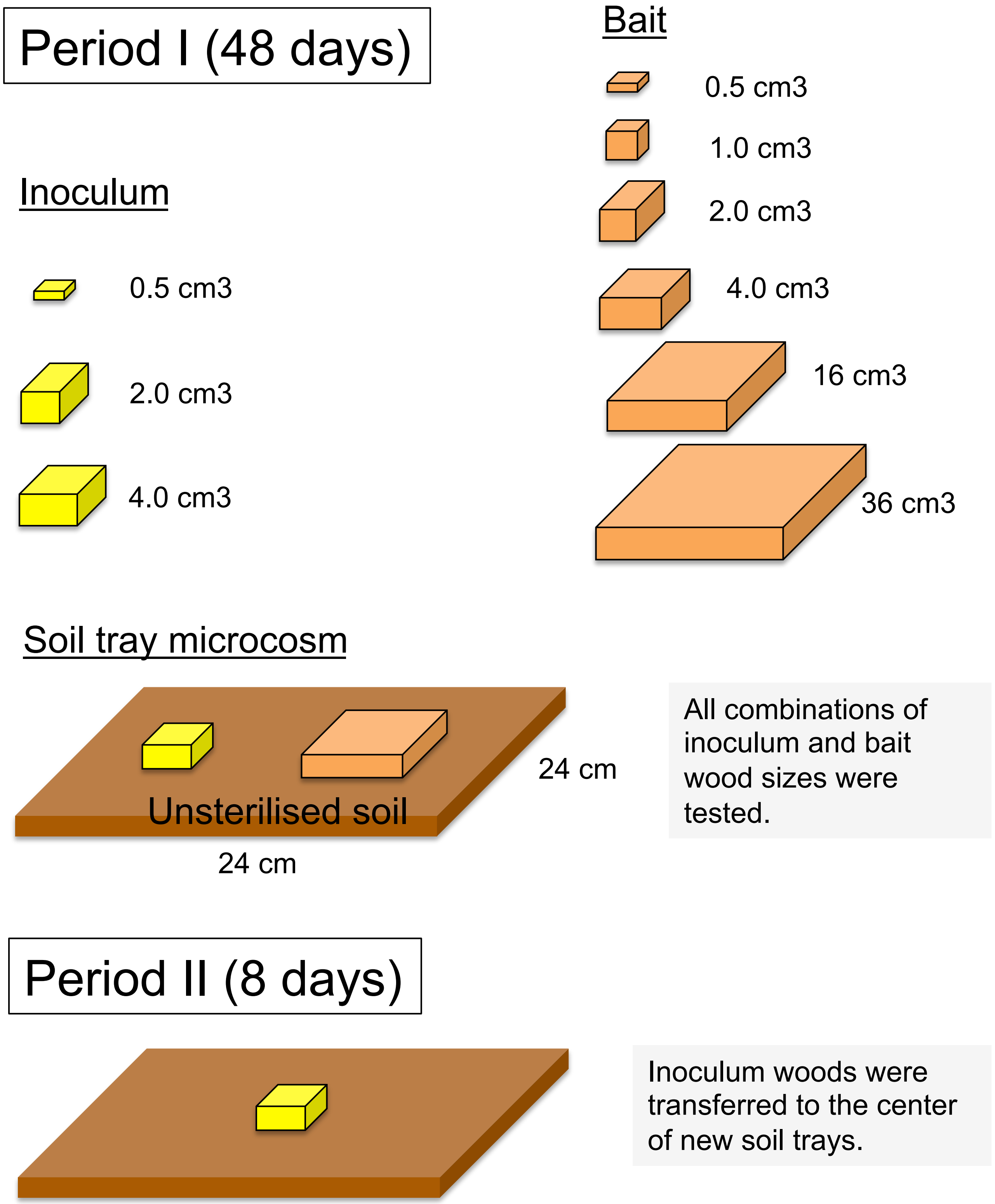


Fig. S1 Schematic diagram of the microcosm design and experimental set up.
