# Supplementary material for: Ecological memory and relocation decisions in fungal mycelial networks: responses to quantity and location of new resources
Source: ISME J. 2019 Oct 18;14(2):380–8. doi: 10.1038/s41396-019-0536-3 (PMC6976561; doi:10.1038/s41396-019-0536-3)
Supplement: Supplementary file 2 — Figure S2 [file 41396_2019_536_MOESM2_ESM.pdf]

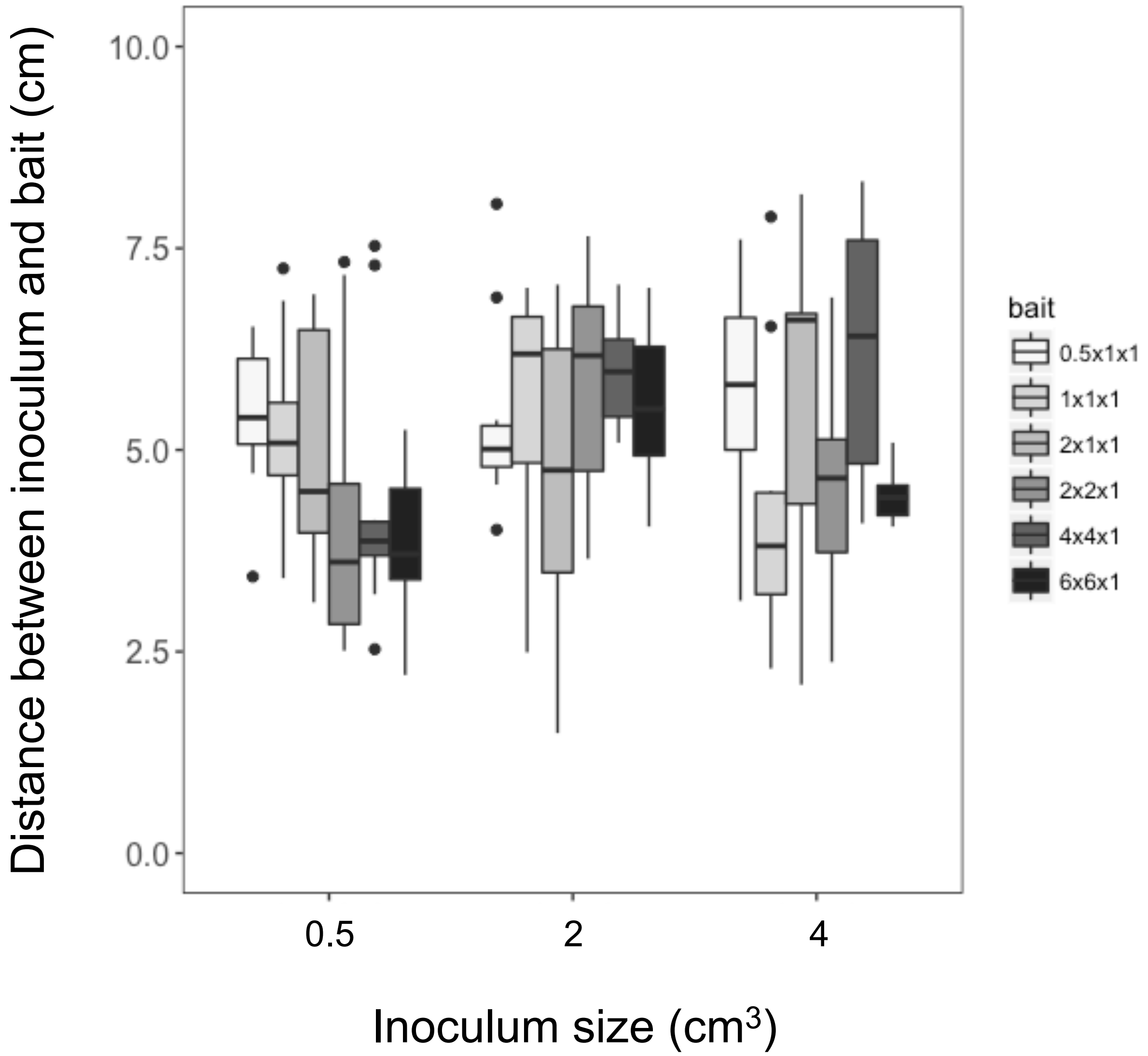

Fig. S2 Distance between inoculum and bait wood blocks in soil microcosms. A generalised linear model (Distance ~ Inoculum size + Bait size, family=gaussian) showed that inoculum and bait sizes both have significantly ($P < 0.05$) associated with the distance.
