# Supplementary material for: Ecological memory and relocation decisions in fungal mycelial networks: responses to quantity and location of new resources
Source: ISME J. 2019 Oct 18;14(2):380–8. doi: 10.1038/s41396-019-0536-3 (PMC6976561; doi:10.1038/s41396-019-0536-3)
Supplement: Supplementary file 3 — Figure S3 [file 41396_2019_536_MOESM3_ESM.pdf]

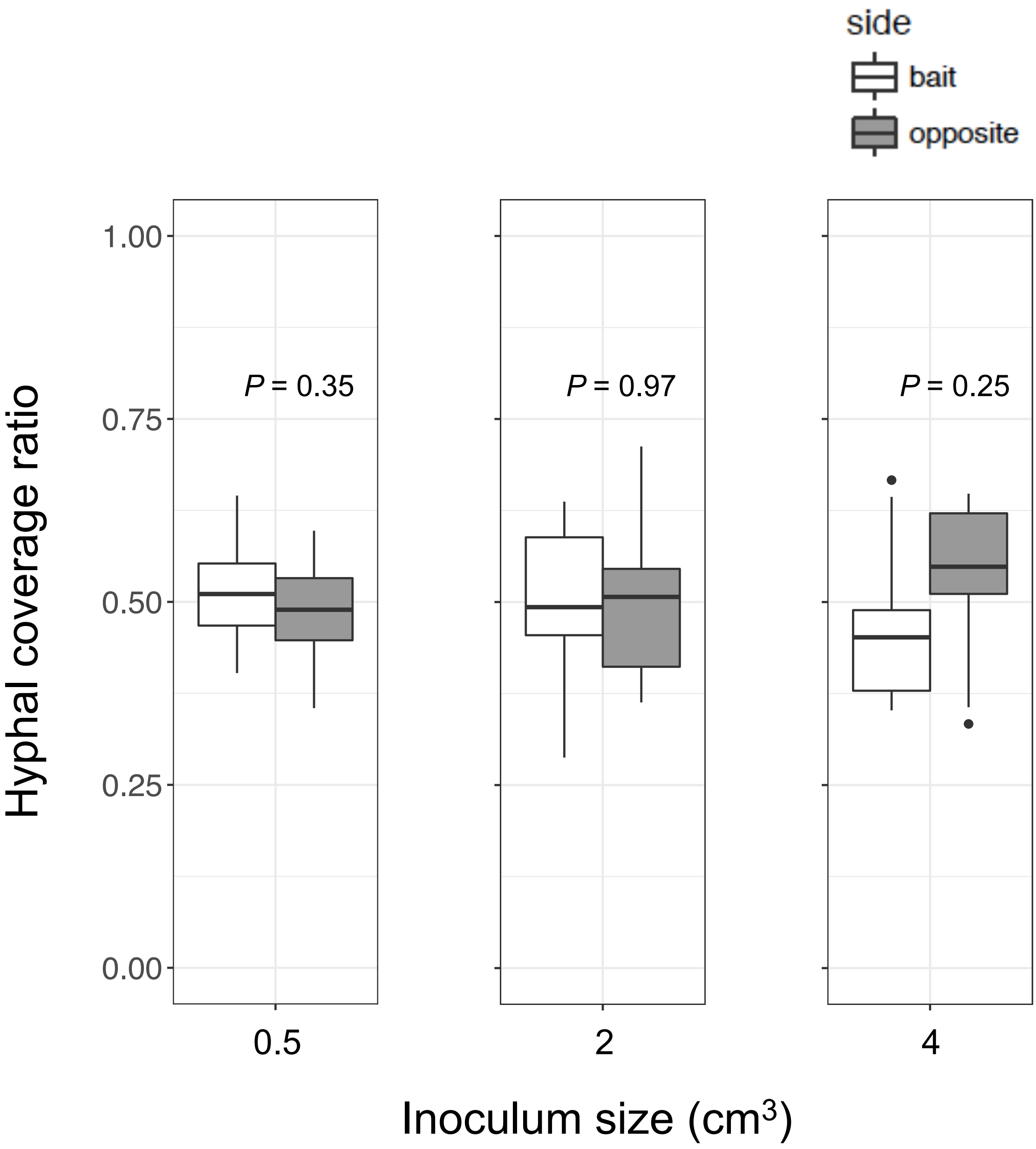

Fig. S3 Hyphal area ratio of bait-side and opposite-side of inoculum on soil microcosm when the baits were added onto the soil. *P*, Wilcoxon rank sum test.
