# Supplementary material for: Ecological memory and relocation decisions in fungal mycelial networks: responses to quantity and location of new resources
Source: ISME J. 2019 Oct 18;14(2):380–8. doi: 10.1038/s41396-019-0536-3 (PMC6976561; doi:10.1038/s41396-019-0536-3)
Supplement: Supplementary file 4 — Figure S4 [file 41396_2019_536_MOESM4_ESM.pdf]

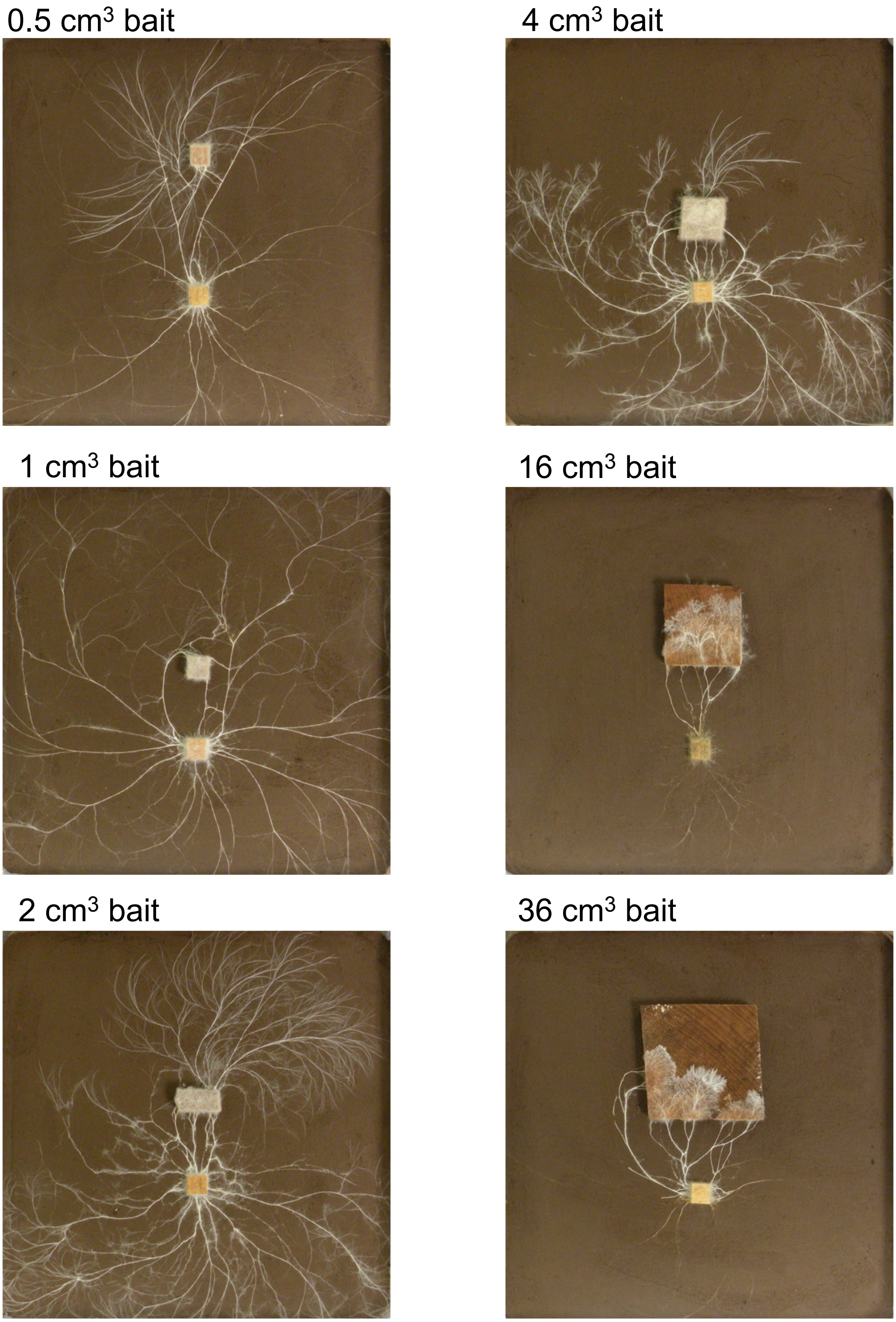

Fig. S4a Typical images of soil microcosms for 0.5 cm^3 inoculum 48 days after coupled with baits of six different sizes. Bottom blocks are inoculum and top blocks are bait.

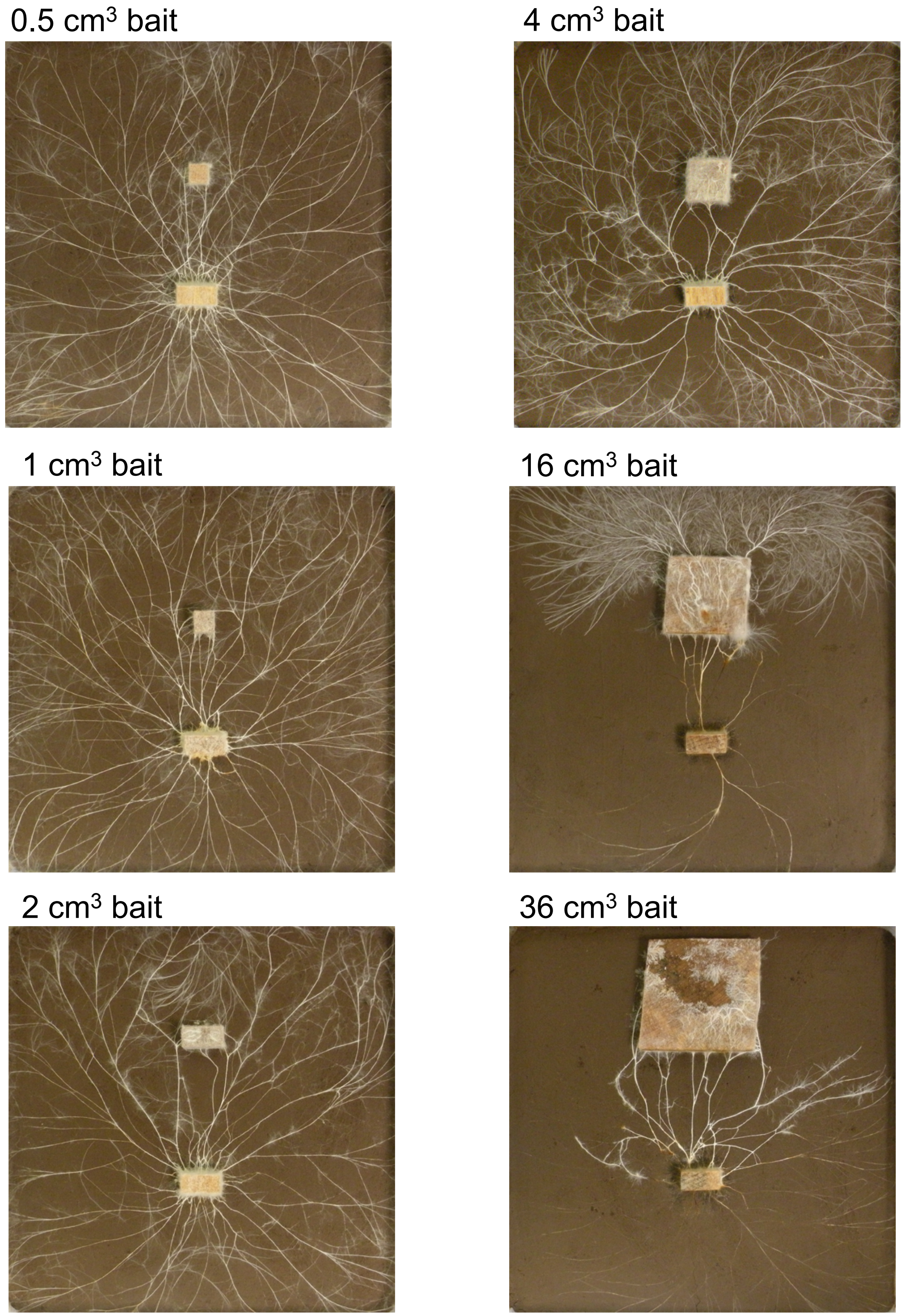

Fig. S4b Typical images of soil microcosms for 2 cm^3 inoculum 48 days after coupled with baits of six different sizes. Bottom blocks are inoculum and top blocks are bait.

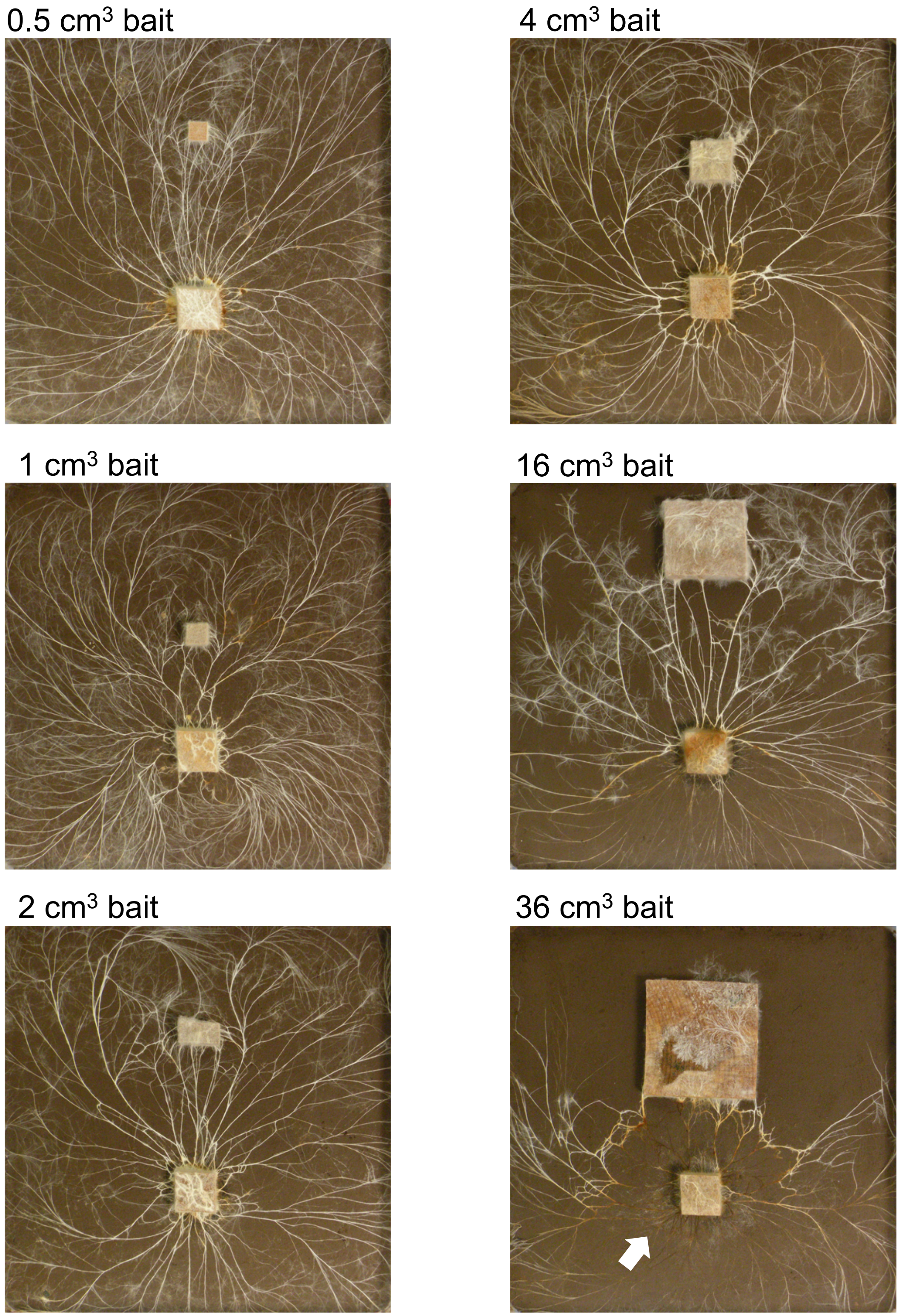

Fig. S4c Typical images of soil microcosms for 4 cm^3 inoculum 48 days after coupled with baits of six different sizes. Bottom blocks are inoculum and top blocks are bait. Arrow indicates dieback of mycelium surrounding inoculum.
